# Depression, Anxiety, and MSQOL-54 Outcomes in RRMS Patients Receiving Fingolimod or Cladribine: A Cross-Sectional Comparative Study

**DOI:** 10.3390/medicina61081409

**Published:** 2025-08-03

**Authors:** Müttalip Özbek, Adalet Arıkanoğlu, Mehmet Ufuk Aluçlu

**Affiliations:** 1 Kiziltepe State Hospital, Department of Neurology, Mardin 47400, Turkey; 2 Department of Neurology, Faculty of Medicine, Dicle University, Diyarbakir 21090, Turkey; neuron472@hotmail.com (A.A.); drufukaluclu21@hotmail.com (M.U.A.)

**Keywords:** multiple sclerosis, fingolimod, cladribine, MSQOL-54, depression, anxiety, EDSS, quality of life

## Abstract

*Background and Objectives*: Multiple sclerosis (MS) is a chronic immune-mediated neurological disorder that primarily affects young adults and is frequently accompanied by psychiatric comorbidities such as depression and anxiety, both of which significantly diminish patients’ quality of life (QoL). This study investigated the effect of two oral disease-modifying therapies (DMTs), fingolimod and cladribine, on mental health and QoL in patients with relapsing-remitting MS (RRMS). The aim of the study was to compare levels of depression, anxiety, and health-related quality of life (HRQoL) in RRMS patients treated with fingolimod or cladribine, and to evaluate their associations with clinical and radiological parameters. *Materials and Methods:* Eighty RRMS patients aged 18 to 50 years with Expanded Disability Status Scale (EDSS) scores of 3.0 or less, no recent disease relapse, and no history of antidepressant use were enrolled. Forty patients were treated with fingolimod and forty with cladribine. Depression and anxiety were assessed using the Hamilton Depression Rating Scale (HDRS) and the Hamilton Anxiety Rating Scale (HARS). QoL was evaluated using the Multiple Sclerosis QoL-54 (MSQOL-54) instrument. Additional clinical data, including MRI-based lesion burden, EDSS scores, age, disease duration, and occupational status, were collected. *Results:* No statistically significant differences were observed between the two groups regarding HDRS and HARS scores (*p* > 0.05). However, patients treated with fingolimod had significantly higher scores in the Energy/Fatigue subdomain (7.55 ± 2.02 vs. 6.56 ± 2.57, *p* = 0.046) and Composite Mental Health (CMH) score (64.73 ± 15.01 vs. 56.00 ± 18.93, *p* = 0.029) compared to those treated with cladribine. No significant differences were found in the independent items of the MSQOL-54. A negative correlation was identified between total lesion load and QoL scores. *Conclusions:* Although fingolimod and cladribine exert comparable effects on depression and anxiety levels, fingolimod may be associated with better mental health outcomes and reduced fatigue in RRMS patients. Furthermore, lesion burden and clinical parameters such as age and EDSS score may independently influence QoL, regardless of the DMT used.

## 1. Introduction

MS is a chronic, immune-mediated neurodegenerative disorder of the central nervous system (CNS) that primarily affects young and middle-aged adults, with a higher prevalence in women. The disease is characterized by multifocal areas of demyelination, inflammation, gliosis, and subsequent axonal degeneration within the brain and spinal cord. These pathological changes result in a wide spectrum of clinical manifestations, including motor, sensory, visual, and cognitive impairments [1]. In addition to its physical burden, MS carries a substantial psychological dimension. Depression and anxiety are among the most common neuropsychiatric comorbidities, affecting approximately 50 percent and 30 percent of patients, respectively. These conditions contribute significantly to poor treatment adherence, social withdrawal, and diminished QoL [2,3,4]. Fatigue, which may occur independently of physical disability, is another major factor that negatively impacts HRQoL in individuals with MS.

RRMS, the most prevalent disease phenotype, is defined by episodes of neurological exacerbation followed by periods of partial or complete remission. Effective therapeutic interventions are essential to reduce relapse frequency and prevent long-term disability. DMTs constitute the cornerstone of RRMS management, aiming to suppress aberrant immune activity and limit CNS inflammation [5]. Among the oral DMTs, fingolimod, a sphingosine-1-phosphate receptor modulator, and cladribine, a purine nucleoside analog that selectively reduces B and T lymphocytes, are commonly prescribed due to their proven efficacy, convenient administration routes, and favorable safety profiles [6,7].

While the immunological and radiological benefits of fingolimod and cladribine have been well established in randomized controlled trials, their effects on neuropsychiatric outcomes and HRQoL remain relatively underexplored. Emerging evidence suggests that DMTs may influence mood, fatigue, and cognitive functioning through mechanisms related to neuroinflammation and CNS penetration [8,9]. Fingolimod, in particular, has been proposed to possess neuroprotective effects, potentially due to its ability to cross the blood–brain barrier, suppress microglial activation, and mitigate hippocampal atrophy. These actions may have beneficial implications for mood and anxiety regulation [10]. In contrast, the neuropsychiatric effects of cladribine are not yet well understood, and clinical data evaluating its influence on mental health remain limited.

Accurate assessment of patient-reported outcomes (PROs) such as QoL requires the use of disease-specific, validated instruments. The MSQOL-54 questionnaire is a comprehensive tool that expands upon the generic SF-36 by incorporating MS-specific domains such as fatigue, cognitive function, and health-related distress. It enables detailed evaluation of both physical and mental well-being and has become widely utilized in research and clinical practice [11].

Despite the relevance of psychological parameters in MS management, direct comparative studies examining the effect of fingolimod and cladribine on depression, anxiety, and HRQoL are lacking. Given the pharmacodynamic differences, varying CNS penetrance, and distinct mechanisms of action of these agents, such investigations are necessary to inform personalized therapeutic decisions that extend beyond traditional measures of relapse rate and physical disability.

**Figure 1:**
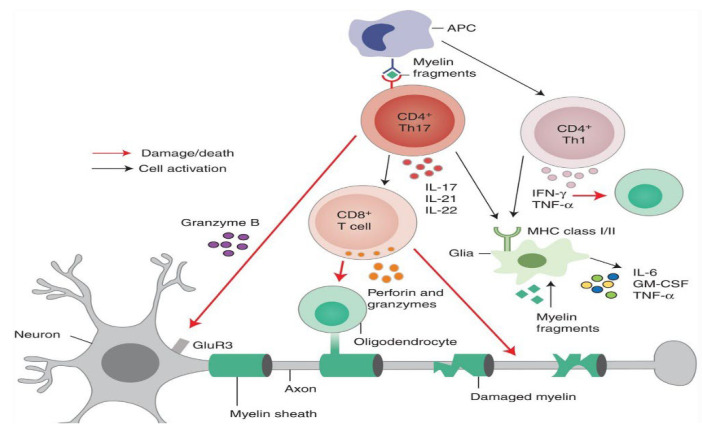
Schematic representation of immune-mediated demyelination in MS. Activated CD4^+^ Th17 and Th1 cells, along with CD8^+^ cytotoxic T cells, release pro-inflammatory cytokines (e.g., IL-17, IFN-γ, TNF-α) and cytotoxic molecules (e.g., granzyme B, perforin), leading to oligodendrocyte injury and myelin sheath degradation. Antigen-presenting cells (APCs) present myelin-derived peptides to T cells, amplifying the autoimmune response. The resulting damage contributes to axonal dysfunction and neurodegeneration characteristic of MS pathology. Figure created using BioRender.com.

Figure 1 provides a simplified schematic representation of the immune-mediated demyelination process in MS, illustrating the involvement of Th1, Th17, and CD8^+^ T cells, as well as their cytotoxic and cytokine-driven effects on oligodendrocytes and axonal structures.

The present study was therefore designed to compare levels of depression, anxiety, and HRQoL in RRMS patients undergoing treatment with either fingolimod or cladribine. Additionally, we aimed to explore the associations between HRQoL outcomes and clinical variables such as EDSS and disease duration, as well as radiological parameters including total lesion burden and lesion localization. We hypothesized that patients receiving fingolimod would exhibit more favorable mental health outcomes and lower levels of fatigue and depression compared to those receiving cladribine.

## 2. Materials and Methods

### 2.1. Study Design and Participants

This cross-sectional, observational, single-center study with prospective patient enrollment was conducted between 15 July 2022 and 15 July 2023 at the Neurology Department of Dicle University Faculty of Medicine, Diyarbakır, Turkey. This study was approved by the Ethics Committee of Dicle University Faculty of Medicine (Meeting No: 254, Date: 9 June 2022), and all procedures performed in this study were in accordance with the ethical standards of the institutional and national research committee, and with the 1964 Helsinki Declaration and its later amendments or comparable ethical standards. Written informed consent was obtained from all individual participants included in the study. A total of 80 patients diagnosed with RRMS, according to the 2017 McDonald criteria, were included. Although patients were recruited prospectively over a one-year period, each participant was evaluated only once at the time of enrollment during a routine outpatient visit. No longitudinal follow-up was performed.

Inclusion criteria were as follows: age between 18 and 50 years; EDSS score ≤ 3.0; at least one year of continuous monotherapy with fingolimod or cladribine; and the ability to independently complete psychometric assessments.

Exclusion criteria included the following: a history of major psychiatric disorders (e.g., bipolar disorder or schizophrenia); relapse within the past 6 months; corticosteroid use within the previous month; or current use of antidepressant, anxiolytic, or antipsychotic medications.

The duration of exposure to current disease-modifying therapy was calculated in months as the time interval between the date of treatment initiation (fingolimod or cladribine) and the date of psychological assessment. Mean treatment durations were 14.6 ± 2.8 months for the cladribine group and 15.3 ± 3.1 months for the fingolimod group.

### 2.2. Treatment Exposure and Assessment Timing

All patients included in the study had received at least one year of continuous monotherapy with fingolimod or cladribine before the time of psychological assessment. For cladribine-treated patients, assessments were performed at least 3 months after the final dose of the second annual treatment cycle, ensuring that the treatment’s immunological effects had stabilized. For fingolimod, assessments were conducted during stable treatment periods without recent initiation or discontinuation.

### 2.3. Clinical and Radiological Data Collection

Demographic variables (age, sex, education level, and employment status) and clinical data (disease duration, EDSS scores) were collected, along with treatment-related information.

All participants underwent brain magnetic resonance imaging, which was evaluated by a neuroradiologist blinded to the clinical information. Lesion load was measured semi-automatically using axial T2-FLAIR sequences. Lesions were categorized by location (periventricular, juxtacortical, and infratentorial), and total lesion volume was calculated in cubic millimeters.

### 2.4. Assessment Instruments

#### 2.4.1. Hamilton Depression and Anxiety Scales

Depression and anxiety were assessed using the HDRS (Omaha, NE, USA) (17 items) and the HARS (Konya, Akşehir) (14 items). Both instruments were developed by Hamilton and are widely used in MS-related clinical research [12].

HDRS scores range from 0 to 53, while HARS scores range from 0 to 56. In both scales, higher scores indicate greater severity of depression or anxiety symptoms.

#### 2.4.2. MSQOL-54 Scale

HRQoL was assessed using MSQOL-54, developed by Vickrey et al. [13].

The MSQOL-54 includes 54 Likert-type items evaluating health over the past four weeks. It combines the core SF-36 with 18 MS-specific items. The scale produces two composite scores: Composite Physical Health (CPH) and CMH.

It also includes 12 subscales: physical function; role limitations due to physical or emotional problems; pain; energy/fatigue; emotional well-being; health perceptions; social function; cognitive function; health distress; sexual function; and overall QoL. In addition, there are two single-item measures: change in health and satisfaction with sexual function.

Higher scores reflect better perceived QoL. In the original study, Cronbach’s alpha was reported as 0.96 for CPH and 0.94 for CMH [14]. In this study, the internal consistency reliability was found to be 0.958 for CPH and 0.906 for CMH.

### 2.5. Statistical Analysis

Statistical analyses were performed using IBM SPSS Statistics for Windows, Version 26.0 (IBM Corp., Armonk, NY, USA). Descriptive statistics were reported as mean ± standard deviation (SD) or frequency and percentage, as appropriate. The normality of continuous variables was tested using the Kolmogorov–Smirnov test. Between-group comparisons were conducted using the independent samples *t*-test or the Mann–Whitney U test for continuous variables, and the chi-square test for categorical variables. Pearson or Spearman correlation coefficients were used to assess relationships between clinical, radiological, and psychometric variables. A two-tailed *p*-value of <0.05 was considered statistically significant. No data imputation was performed; analyses were based on complete cases only.

A post hoc power analysis for the observed between-group difference in CMH score (mean difference: 8.73, SDpooled: 17.1) yielded a statistical power of 73.2% at α = 0.05 (two-tailed), indicating moderate sensitivity to detect group differences.

## 3. Results

### 3.1. Demographic and Clinical Characteristics

A total of 80 RRMS patients were included, with 40 receiving fingolimod and 40 receiving cladribine. The groups were comparable in mean age (34.1 ± 7.5 vs. 33.4 ± 7.3 years; *p* = 0.672), sex distribution (female: 65% vs. 62.5%; *p* = 0.804), education, and disease duration (*p* > 0.05). EDSS scores also showed no significant difference (*p* > 0.05), confirming baseline comparability (Table 1). However, occupational status differed significantly (χ^2^ = 11.635; *p* = 0.033), with a higher proportion of unemployed and civil servants in the cladribine group.

**Figure 2:**
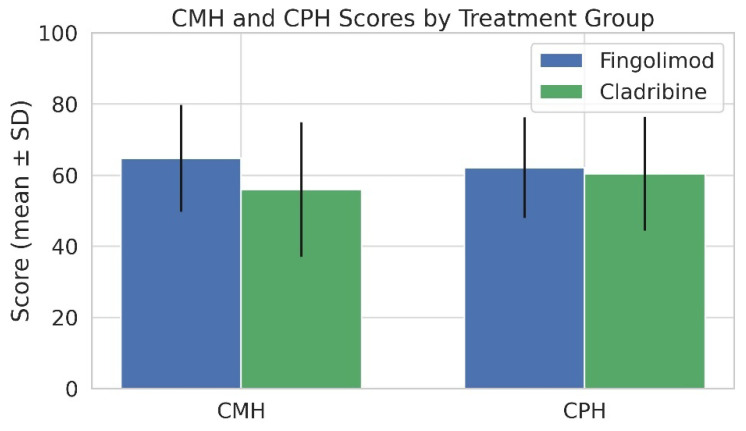
Comparison of mean CMH and CPH scores derived from the MSQOL-54 between RRMS patients treated with fingolimod and cladribine. Error bars represent standard deviations (SD). Patients using fingolimod exhibited significantly higher CMH scores (64.73 ± 15.01) than those receiving cladribine (56.00 ± 18.93) (*p* = 0.029, independent samples *t*-test), suggesting better perceived mental well-being. No significant difference was found between the groups in terms of CPH scores (68.93 ± 14.16 vs. 61.15 ± 19.75; *p* > 0.05, independent samples *t*-test).

In the comparison of composite QoL scores between the two treatment groups, patients receiving fingolimod exhibited significantly higher CMH scores than those receiving cladribine (64.73 ± 15.01 vs. 56.00 ± 18.93, *p* = 0.029), suggesting improved perceived mental well-being. In contrast, no statistically significant difference was found in CPH scores between the fingolimod and cladribine groups (63.28 ± 17.45 vs. 61.87 ± 17.65, *p* > 0.05). These findings indicate that fingolimod may be associated with better mental health outcomes in RRMS patients, while both treatments exert comparable effects on physical HRQoL (Figure 2).

### 3.2. Occupational Status and Psychosocial Implications

A significant difference was observed in occupational status between the treatment groups, with more unemployed and civil servant patients in the cladribine group, while fingolimod users had higher rates of homemakers, students, and workers. Although direct statistical analyses correlating occupational status with psychological or QoL scores were not conducted, these demographic disparities may influence the observed differences in lesion burden and QoL. Future research should explore the effect of occupational status on psychological well-being and HRQoL in MS patients.

### 3.3. Depression and Anxiety Scores

Depression and anxiety scores measured by HDRS and HARS did not differ significantly between groups (*p* > 0.05) (Table 2). The distribution of depression and anxiety severity categories (Normal, Mild, Moderate, Severe) is visualized in Table 2, indicating comparable psychological symptom burdens across treatments.

**Figure 3:**
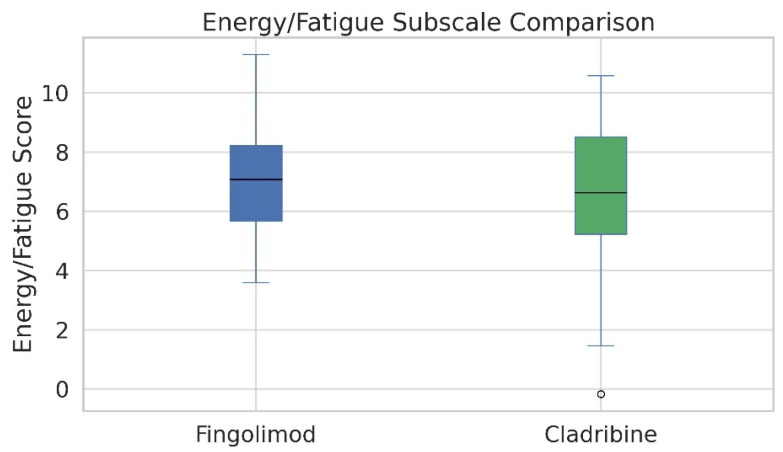
Boxplot illustrating Energy/Fatigue subscale scores from the MSQOL-54 questionnaire among RRMS patients treated with fingolimod and cladribine. The box represents the interquartile range (IQR), the horizontal line indicates the median, and the whiskers show minimum and maximum values excluding outliers. One outlier is visible in the cladribine group. The fingolimod group demonstrated significantly higher Energy/Fatigue scores (mean ± SD: 7.55 ± 2.02) compared to the cladribine group (6.56 ± 2.57), suggesting reduced perceived fatigue (*p* = 0.046, Mann–Whitney U test).

When comparing the Energy and Fatigue subscale scores of the MSQOL-54 between treatment groups, patients using fingolimod reported significantly higher levels of perceived energy than those treated with cladribine. The mean score for the fingolimod group was 7.55 with a standard deviation of 2.02, while the cladribine group had a mean score of 6.56 and a standard deviation of 2.57. This difference reached statistical significance (*p* = 0.046), indicating that individuals receiving fingolimod may experience lower levels of fatigue in daily life. The distribution of scores, as depicted in the boxplot, further supports this finding by illustrating a higher central tendency and narrower interquartile range in the fingolimod group (Figure 3).

### 3.4. MSQOL-54 Scores

**Figure 4:**
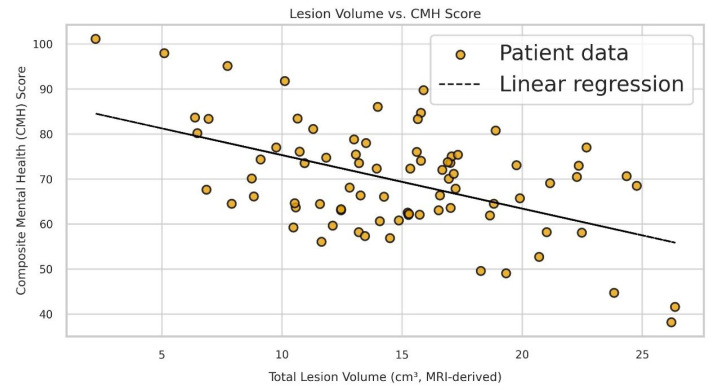
Scatter plot illustrating the inverse correlation between total MRI-derived lesion volume (cm^3^) and CMH scores among RRMS patients (n = 80). Each dot represents an individual patient’s data. The overlaid regression line reflects a statistically significant negative correlation (r = −0.312, *p* = 0.021), computed using Spearman’s rank correlation coefficient. This result indicates that higher total lesion burden is significantly associated with reduced CMH scores. Analyses were performed using IBM SPSS Statistics v26.0.

**Figure 5:**
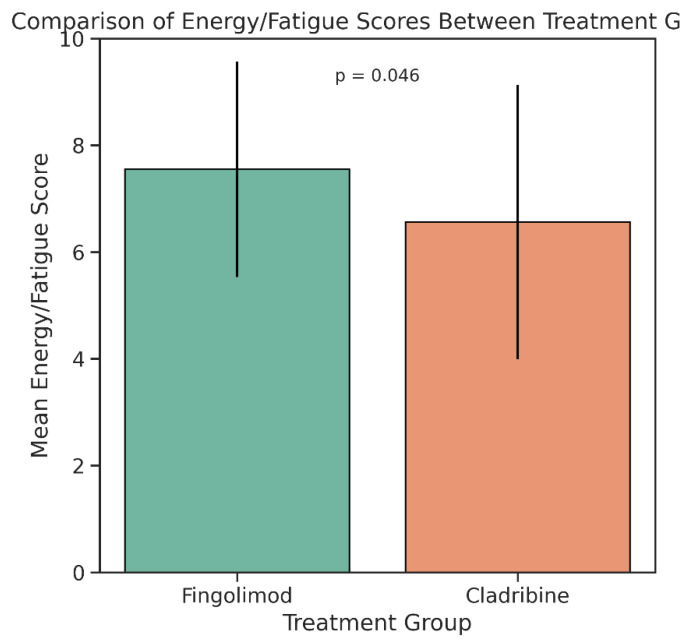
Comparison of Energy/Fatigue subscale scores between RRMS patients treated with fingolimod and cladribine. The mean score was significantly higher in the fingolimod group (7.55 ± 2.02) compared to the cladribine group (6.56 ± 2.57), indicating better perceived energy levels among patients receiving fingolimod. The between-group difference was statistically significant (*p* = 0.046), based on an independent samples *t*-test. Error bars represent standard deviations (SD). Statistical analysis was conducted using IBM SPSS Statistics Version 26.0.

**Figure 6:**
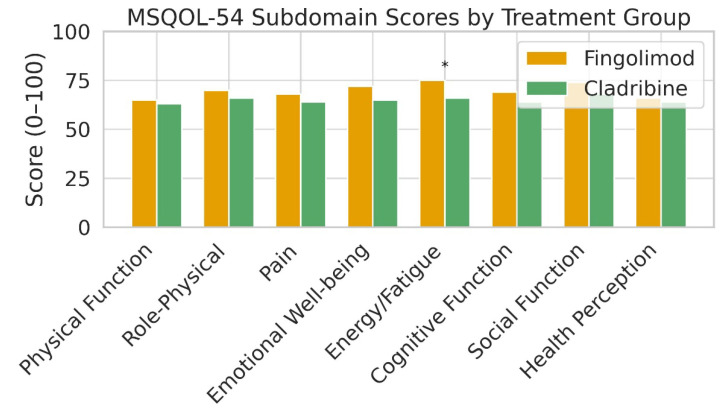
Bar chart illustrating the mean scores of eight MSQOL-54 subdomains in RRMS patients treated with fingolimod (n = 40) or cladribine (n = 40). Patients receiving fingolimod demonstrated significantly higher scores in the Energy/Fatigue domain (*p* = 0.046), denoted by an asterisk (*). All other subdomains—Physical Function, Role-Physical, Pain, Emotional Well-being, Cognitive Function, Social Function, and Health Perception—showed no statistically significant differences between treatment groups (*p* > 0.05). Statistical comparisons were conducted using independent samples *t*-tests. Analyses were performed in IBM SPSS Statistics Version 26.0. The results indicate that fingolimod may provide a specific advantage in reducing fatigue-related QoL impairments among RRMS patients.

Fingolimod users had significantly higher CMH scores than cladribine users (64.73 ± 15.01 vs. 56.00 ± 18.93; *p* = 0.029), while CPH scores showed no significant difference (62.14 ± 14.12 vs. 60.43 ± 16.01; *p* > 0.05) (Figure 4). Additionally, the Energy/Fatigue subscale was significantly higher in the fingolimod group (7.55 ± 2.02 vs. 6.56 ± 2.57; *p* = 0.046) (Figure 5). No significant differences were observed in other MSQOL-54 subdomains (Table 3 and Figure 6).

In the MSQOL-54 scoring system, higher scores in the ‘Role limitations due to emotional problems’ domain indicate fewer limitations, which is consistent with the higher CMH scores in the fingolimod group.

### 3.5. Correlation Analyses

**Figure 7:**
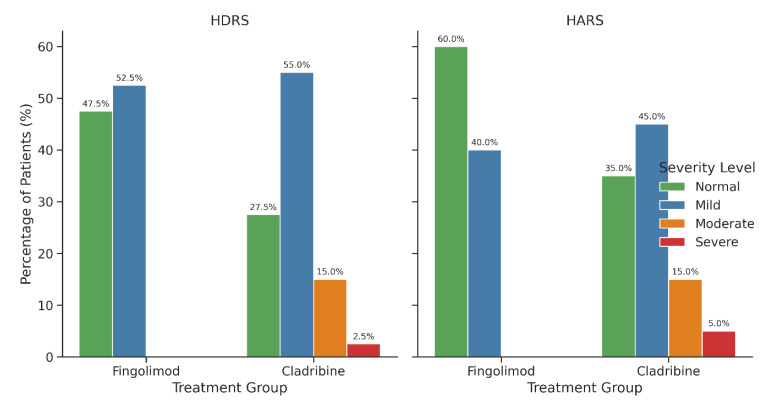
Distribution of depression and anxiety severity levels among RRMS patients treated with fingolimod and cladribine. Severity categories (Normal, Mild, Moderate, and Severe) are presented as percentages within each treatment group based on HDRS (**left** panel) and HARS (**right** panel) scores. In the fingolimod group, 47.5 percent of patients were classified as Normal and 52.5 percent as Mild in HDRS, while in the cladribine group, 27.5 percent were Normal, 55.0 percent Mild, 15.0 percent Moderate, and 2.5 percent Severe. Regarding anxiety severity, 60.0 percent of fingolimod patients fell within the Normal range and 40.0 percent as Mild, whereas cladribine users showed 35.0 percent Normal, 45.0 percent Mild, 15.0 percent Moderate, and 5.0 percent Severe levels. Although chi-square tests revealed no statistically significant differences in severity distributions between treatment groups (*p* greater than 0.05), the cladribine group exhibited higher proportions of moderate to severe psychiatric symptoms. These findings emphasize the need for continuous psychological assessment in RRMS management, regardless of disease modifying therapy.

**Figure 8:**
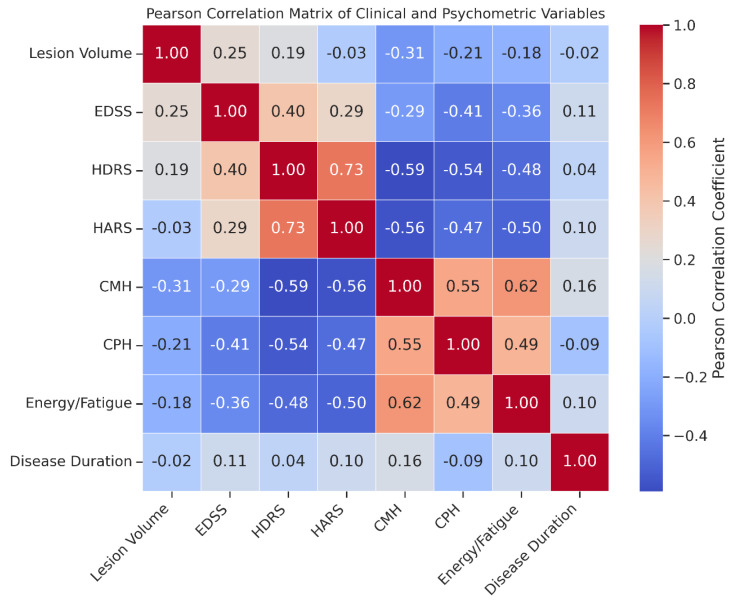
Pearson correlation matrix illustrating the interrelationships among clinical (lesion volume, EDSS, and disease duration) and psychometric variables (HDRS, HARS, CMH, CPH, and Energy/Fatigue) in RRMS patients (n = 80). A significant negative correlation was observed between EDSS and both CMH (r = −0.29, *p* = 0.031) and CPH (r = −0.41, *p* = 0.004), indicating that greater disability is associated with worse mental and physical health perceptions. Strong positive correlation was found between HDRS and HARS scores (r = 0.73, *p* < 0.001), while both depression and anxiety levels were inversely correlated with CMH (r = −0.59 and −0.56, respectively; both *p* < 0.001). Moreover, CMH demonstrated a significant positive correlation with Energy/Fatigue (r = 0.62, *p* < 0.001), emphasizing the strong link between mental health and fatigue perception. These results underscore the interplay between disease burden, psychological status, and QoL in RRMS.

Figure 6 depicts a significant inverse correlation between total lesion volume and CMH scores (r = −0.312, *p* = 0.021), suggesting greater lesion burden is associated with poorer mental HRQoL. The distribution of depression and anxiety severity categories (Normal, Mild, Moderate, Severe) among RRMS patients is presented in Figure 7. Both treatment groups predominantly consisted of patients within the Normal and Mild severity levels, with fewer patients classified as Moderate or Severe. These findings indicate that fingolimod and cladribine have comparable effects on the severity of psychiatric symptoms. Clinically, this suggests equivalent efficacy of both therapies in managing depression and anxiety in RRMS patients. Although statistical significance was not reached, the monitoring of psychological symptoms remains crucial during treatment selection to optimize patient outcomes and improve QoL. Objective assessment of depression and anxiety levels can guide clinicians in tailoring comprehensive management strategies. Figure 8 presents the Pearson correlation matrix detailing relationships among lesion volume, EDSS, disease duration, and psychological scales (HDRS, HARS, CMH, CPH, Energy/Fatigue), with notable correlations including EDSS vs. CMH (r = −0.29) and EDSS vs. CPH (r = −0.41).

## 4. Discussion

This study provides a comprehensive evaluation of psychological well-being and quality of life in patients with RRMS treated with fingolimod or cladribine. Our findings showed that while both groups had comparable levels of depression and anxiety, patients treated with fingolimod demonstrated significantly higher scores in the Energy/Fatigue domain and CMH index of the MSQOL-54. These results suggest potential advantages of fingolimod in enhancing mental well-being and fatigue-related quality of life. By integrating clinical variables, radiological findings, and psychometric scales, this study contributes to a more individualized and patient-centered approach in RRMS management.

Studies comparing individuals with MS to both healthy populations and other patient groups have consistently shown that MS patients experience a lower QoL compared to both comparison groups [14,15,16,17,18,19]. In Vickrey’s original study reporting the results of the MSQOL-54, MS patients were compared with healthy controls matched by age and gender. The study demonstrated that MS patients had significantly lower scores across all dimensions of the scale. The differences in QoL scores between MS patients and healthy individuals ranged from 8 to 48 points with an average difference of 25 to 30 points. In our study CPH score was 65.04 ± 17.52 and CMH score was 60.37 ± 17.53 compared to 48.6 ± 20.3 and 62.9 ± 20.7, respectively, in the original study [13]. These higher scores in our cohort were attributed to the lower EDSS levels of our patients since the original cohort included individuals with more advanced disability.

When analyzing MSQOL-54 main and subdimension scores in relation to demographic factors, we found that female patients had significantly higher scores in the physical health, pain, social function, and sexual function subdomains of the CPH dimension compared to male patients. While Visser et al. [14] did not specifically analyze gender differences in MSQOL-54 subscales, their multicenter study highlighted substantial variability in HRQOL scores and emphasized the influence of clinical factors such as disability status and disease course. No significant associations were found between gender and the independent MSQOL-54 items assessing change in health and satisfaction with sexual function.

In our study, patients who had been treated with fingolimod or cladribine for at least three months were included. No significant differences were observed between the two treatment groups regarding age EDSS scores or disease duration indicating that the groups were demographically and clinically comparable. Additionally, no significant differences were found in HDRS and HARS scores between the two groups suggesting similar levels of depression and anxiety. The proportion of patients with anxiety and depression was 52.5 percent in the cladribine group and 55 percent and 57.5 percent, respectively, in the fingolimod group. These rates are substantially higher than those reported in the general population. In a study on MS patients using interferon beta (IFN beta), although no cases of major depressive disorder were reported, severe mood disturbances and occasional suicidal ideation were observed [20]. Another study from Brazil reported increased rates of depression and anxiety among MS patients treated with fingolimod and IFN beta. In a comparative study of cytokine levels, patients using IFN beta had a higher risk of depression than those on fingolimod therapy [21].

To our knowledge no previous study has directly compared the oral use of fingolimod and cladribine in terms of psychological outcomes as conducted here. In 2017 the SF 36 questionnaire was used in the OPERA II double blind active comparator study, which compared ocrelizumab with IFN beta 1a. The physical composite score at 24 months was significantly higher in the ocrelizumab group [22]. In another comparative study of dimethyl fumarate, IFN beta, and glatiramer acetate, patients treated with dimethyl fumarate reported better QoL than those on the other therapies [23].

In our analysis, the energy and fatigue subscale and overall CMH score were higher in patients treated with fingolimod compared to those treated with cladribine. This finding may be related to a greater total lesion burden in the cladribine group particularly involving the juxtacortical and cortical areas. Fingolimod may contribute to improved mental health outcomes through its central neuroprotective and anti-inflammatory mechanisms. As a sphingosine 1 phosphate (S1P) receptor modulator, fingolimod crosses the blood–brain barrier and binds to S1P receptors expressed on microglia and astrocytes. This binding reduces microglial activation, suppresses pro-inflammatory cytokine release, and attenuates hippocampal atrophy, all of which are processes implicated in mood regulation and cognitive functioning. In contrast, cladribine is a purine nucleoside analog that selectively depletes memory B cells and subsets of T lymphocytes involved in chronic neuroinflammation. Emerging evidence indicates that cladribine can penetrate the central nervous system, lower neurofilament light chain levels in the cerebrospinal fluid, and exert effects on slowly expanding gray matter lesions. Although both agents primarily modulate peripheral immune activity, their distinct central nervous system profiles may help explain the differences observed in mental health-related QoL and fatigue scores between the treatment groups in this study [10,24,25].

Previous research has demonstrated that QoL measures in MS patients are closely associated with white matter lesion load and brain atrophy observed on MRI [26]. Although we did not conduct detailed statistical comparisons based on lesion localization, a general inverse association was observed between overall lesion burden including its topographical distribution and HRQoL scores. This trend aligns with existing hypotheses that certain CNS lesion sites such as cortical or brainstem regions may have a greater effect on neuropsychiatric symptoms and perceived well-being in patients with RRMS. Future studies involving volumetric and topographical MRI analyses in larger cohorts will be important to further investigate these associations.

In our cohort, 62.5 percent of patients experienced mild moderate or severe depression and 67.5 percent had minor or major anxiety. In comparison, a US-based study reported a lifetime prevalence of depression in the general population of 16 percent [27], with anxiety prevalence reported at 27.3 percent [28]. The lifetime prevalence of depression in MS patients has been estimated at 30 to 40 percent [29,30] while anxiety prevalence ranges from 35 to 65 percent [31]. The elevated rates of depression and anxiety in our study are likely multifactorial but may have been exacerbated by the 7 February 2023 earthquake which has been referred to as the Disaster of the Century. Given that our study was conducted in Diyarbakır Province between 15 July 2022, and 15 July 2023, a region heavily affected by the disaster, this contextual factor may have contributed significantly to the psychological burden observed in our participants. Since the study continued until July 2023, a substantial number of patients were assessed after the 7 February 2023 earthquake. Therefore, it is reasonable to consider this disaster as a potential psychosocial stressor influencing anxiety and depression scores.

The recent literature continues to emphasize the importance of evaluating both physical and psychological dimensions of HRQoL in patients with RRMS, especially those receiving DMTs such as cladribine or fingolimod. In our study, although the overall depression and anxiety levels were relatively high in both treatment groups, MSQOL-54 CPH and CMH scores indicated preserved QoL, particularly among patients receiving fingolimod. These findings partially align with those of the CLARIFY-MS trial, where Brochet et al. demonstrated that cladribine tablets significantly improved both physical and mental health composite scores over a 24-month period regardless of prior treatment status [32]. Furthermore, an extension of the CLARIFY-MS study reported by Smyk confirmed that these improvements were sustained over a 4-year period, with stable cognition and strong correlations between MSQOL-54 scores and cognitive function as assessed by BICAMS, particularly in treatment-naïve individuals [33]. These long-term data highlight the multidimensional therapeutic benefits of CladT, not only on physical disability and relapse reduction but also on mental resilience and cognitive preservation.

Importantly, our findings regarding elevated depression and anxiety rates—despite ongoing treatment—are also consistent with recent studies that underscore the profound effect of psychological distress on perceived QoL in MS patients. For instance, Yalachkov et al. reported that depression and psychological distress were among the strongest predictors of lower EQ-5D indices, independent of cognitive or motor impairment, in both RRMS and progressive MS populations [34]. Similarly, Gómez-Melero et al. noted that cognitive dysfunction, although impactful, often interacts with anxiety and mood disturbances, making it difficult to isolate its independent contribution to HRQoL [35]. This complex interplay underscores the necessity of incorporating comprehensive psychological screening and support into the long-term management of MS, especially in post-disaster settings such as the 2023 earthquake that affected our study cohort.

From a demographic standpoint, our observation that female patients had higher scores in several CPH subdomains appears to contradict findings from Sabanagic-Hajric et al., who showed significantly lower HRQoL scores in women, especially on the pain scale, and emphasized the detrimental effect of aging on physical health dimensions [36]. This discrepancy might reflect regional sociocultural factors, sample differences, or the timing of evaluation relative to disease onset and treatment initiation. Nonetheless, both studies underscore the importance of stratifying HRQoL data by gender and age to better inform personalized treatment strategies.

Another dimension of emerging importance is the integration of PROs and wearable technology in real-world settings. A prospective observational study protocol from Italy by Borriello et al. aims to assess the longitudinal effects of cladribine on physical and psychological PROs, using tools such as the MSIS-29 and HADS, while also collecting activity data via wearable devices [37]. Such innovative approaches could offer more granular insights into daily functioning and treatment efficacy, particularly in populations similar to ours, where conventional clinical measures such as EDSS might not fully capture the psychosocial burden of disease.

Lastly, a recent review by Buttari et al. explored the CNS effects of cladribine, highlighting its ability to modulate both acute and chronic inflammation, penetrate the blood–brain barrier, reduce CSF biomarkers such as neurofilament light chain, and minimize slowly expanding lesions in patients with predominant gray matter pathology [38]. These biological effects not only support the clinical efficacy of cladribine in reducing disease progression but also may explain the improved cognitive outcomes reported in long-term studies. Although our own MRI-based lesion burden data were limited, our finding that fingolimod-treated patients had better fatigue and CMH scores could reflect differences in lesion topography or immunomodulatory profiles between the two treatments.

This study offers a unique contribution by directly comparing the psychological and QoL outcomes in RRMS patients receiving fingolimod or cladribine. The use of validated psychometric instruments such as the MSQOL-54, HDRS, and HARS ensures a robust assessment of both mental health and HRQoL. Including MRI findings on lesion distribution provides additional insight into the neuroanatomical context of PROs. Furthermore, the demographic and clinical comparability between treatment groups enhances internal validity and minimizes potential confounding. The timing of data collection in a post-disaster setting also adds a valuable dimension, capturing psychological responses under extraordinary stress conditions and contributing a novel perspective to the existing literature.

The cross-sectional design limits the ability to establish causal relationships between treatment type, lesion distribution, and psychological outcomes. The absence of volumetric imaging data, such as measurements of brain atrophy, restricts the depth of neuroimaging analysis. Similarly, the lack of standardized cognitive performance assessments prevents a comprehensive understanding of cognitive function in relation to mental health scores. Another limitation stems from the study period, which coincided with the aftermath of a major earthquake. This external stressor may have influenced depression and anxiety levels among participants, potentially limiting the generalizability of the findings to MS populations in unaffected regions.

Future research should employ longitudinal designs to examine the evolving effect of fingolimod and cladribine on mental health, cognition, and QoL. The integration of standardized cognitive test batteries, including BICAMS, alongside PROs and neuroimaging biomarkers, will be essential for capturing the full scope of therapeutic effects. The use of wearable technology and mobile applications may offer real-time monitoring of fatigue, sleep, and emotional fluctuations, providing more ecologically valid data on treatment response. Moreover, future studies should investigate the role of psychosocial factors, such as social support and trauma exposure, in modulating treatment outcomes. Identifying predictive markers for psychological vulnerability or resilience may also allow for more personalized care strategies aimed at optimizing both mental well-being and treatment adherence in RRMS patients.

## 5. Conclusions

This study provides real-world evidence comparing the psychological well-being and HRQoL in patients with RRMS who were treated with fingolimod or cladribine. Despite the relatively high prevalence of depression and anxiety, reported in 57.5 percent and 55 percent of fingolimod-treated patients and 52.5 percent of cladribine-treated patients, both groups demonstrated preserved HRQoL. The mean CPH score was 65.04 with a standard deviation of 17.52, and the CMH score was 60.37 with a standard deviation of 17.53, indicating relatively stable psychosocial function. Patients receiving fingolimod had higher scores in the energy and fatigue subscale and overall mental health domain, which may reflect differences in radiological burden, particularly the distribution of lesions in the juxtacortical and cortical areas.

There were no significant differences between the two treatment groups in terms of age, EDSS scores, or disease duration, supporting the comparability of the study population. These findings suggest that both fingolimod and cladribine are effective in maintaining PROs, although fingolimod may confer additional advantages in fatigue and mental health dimensions. The high burden of psychiatric symptoms observed across both groups is likely multifactorial, with contextual factors such as the 2023 earthquake potentially contributing to the elevated levels of distress.

These results emphasize the importance of incorporating mental health evaluation into routine MS care. Further longitudinal studies integrating volumetric imaging, cognitive testing, and biomarker analysis are needed to confirm these associations and to identify predictors of resilience and treatment response in diverse clinical settings.

## Figures and Tables

**Table 1:** Demographic, clinical, and radiological characteristics of RRMS patients.

**Continuous Variables**		
Variable	Mean ± SD	Min–Max
Age (years)	35.03 ± 8.50	19–49
Disease duration (years)	8.25 ± 5.21	1–21
Duration of current DMT (months)	Cladribine: 14.6 ± 2.8 Fingolimod: 15.3 ± 3.1	12–22
Total lesion amount	2.01 ± 0.68	1–4
EDSS	1.37 ± 0.78	0–2.5
**Categorical Variables**		
Category		n (%)
Gender		
Female		58 (72.5%)
Male		22 (27.5%)
Drug		
Cladribine		40 (50%)
Fingolimod		40 (50%)
Employment status		
Unemployed		15 (18.8%)
Housewife		41 (51.2%)
Officer		13 (16.3%)
Worker		5 (6.3%)
Student		6 (7.5%)
Lesion location		
PV		16 (20.0%)
PV + BS		4 (5.0%)
PV + MS		43 (53.8%)
PV + Cerebellum		2 (2.5%)
PV + SC + BS		5 (6.3%)
PV + BS + Cerebellum		7 (8.8%)
BS + SC + Cerebellum		1 (1.3%)
PV + BS + SC + Cerebellum		2 (2.5%)
Cortical/Juxtacortical		42 (55.0%)

BS: brain stem; EDSS: Expanded Disability Status Scale score; PV: periventricular; SC: spinal cord.

**Table 2:** Hamilton Depression and Anxiety Scales and MSQOL-54 subscores.

Variable	X ± SD/n (%)	Min–Max/Total
Hamilton Depression Rating Scale		
No	30 (37.5%)	
Mild	44 (55.0%)	
Moderate	5 (6.3%)	
Severe	1 (1.3%)	
Hamilton Anxiety Rating Scale		
No	26 (32.5%)	
Minor	43 (53.8%)	
Major	11 (13.8%)	
Total	80	100%
Composite Physical Health	65.04 ± 17.52	25.6–97.3
Physical health	13.56 ± 4.14	4.3–17.0
Role limitations due to physical problems	7.91 ± 4.89	0–12.0
Pain	7.26 ± 2.23	2.6–11.0
Energy	7.06 ± 2.35	1.4–12.0
Social function	7.86 ± 2.05	2.0–12.0
Health perceptions	9.90 ± 3.10	4.3–17.0
Health distress	6.68 ± 2.21	2.2–11.0
Sexual function	5.03 ± 2.10	0–8.0
Composite Mental Health	60.37 ± 17.53	24.9–94.0
Role limitations due to emotional problems	16.72 ± 9.10	0–24.0
Emotional well-being	17.88 ± 4.80	7.7–29.0
Health distress	6.72 ± 2.31	2.2–14.0
Cognitive function	6.98 ± 3.43	1.5–14.3
Overall quality of life	12.13 ± 3.80	1.8–18.0
Single-item Measures		
Change in health	68.44 ± 20.18	0–100
Satisfaction with sexual function	58.44 ± 28.66	0–100

**Table 3:** MSQOL-54 subscale scores by treatment group.

Subscale	Drug	N	Mean ± SD	Median	Min–Max	*p*-Value
Composite Physical Health	Cladribine	40	61.15 ± 19.75	62.7	25.6–97.3	0.063
Physical Health	Cladribine	40	12.70 ± 4.60	13.6	4.2–17.0	0.101
Role Limitations due to Physical Problems	Cladribine	40	7.05 ± 5.20	6	0–12	0.164
Pain	Cladribine	40	7.12 ± 2.47	7	2.5–11	0.533
Energy	Cladribine	40	6.56 ± 2.57	6.4	1.4–12	* 0.046
Social Function	Cladribine	40	7.73 ± 2.30	7	2.0–12	0.563
Health Perceptions	Cladribine	40	9.41 ± 3.28	8.5	4.2–17.0	0.064
Health Distress (CPH)	Cladribine	40	6.27 ± 2.31	6.6	2.2–11.0	0.138
Sexual Function	Cladribine	40	4.83 ± 2.16	5.3	0–8	0.248
Composite Mental Health	Cladribine	40	56.00 ± 18.93	58.1	24.9–93.2	* 0.029
Role Limitations due to Emotional Problems	Cladribine	40	14.20 ± 10.00	16	0–24	* 0.021
Emotional Well-being	Cladribine	40	17.10 ± 5.04	17.4	7.6–27.8	0.213
Health Distress (CMH)	Cladribine	40	6.34 ± 2.51	6.6	2.2–14	0.143
Cognitive Function	Cladribine	40	7.08 ± 3.45	6.7	1.5–14.2	0.783
Overall Quality of Life	Cladribine	40	11.46 ± 4.13	11.5	1.8–18.0	0.106
Composite Physical Health	Fingolimod	40	68.93 ± 14.16	67.7	42.5–95.0	
Physical Health	Fingolimod	40	14.42 ± 3.48	17	4.2–17.0	
Role Limitations due to Physical Problems	Fingolimod	40	8.77 ± 4.47	12	0–12	
Pain	Fingolimod	40	7.41 ± 1.98	7.9	3.3–11	
Energy	Fingolimod	40	7.55 ± 2.02	7.9	1.9–11.5	
Social Function	Fingolimod	40	8.00 ± 1.80	8	4.0–12	
Health Perceptions	Fingolimod	40	10.40 ± 2.85	9.3	5.1–16.1	
Health Distress (CPH)	Fingolimod	40	7.10 ± 2.05	6.6	2.2–11.0	
Sexual Function	Fingolimod	40	5.24 ± 2.03	5.3	1.3–8.0	
Composite Mental Health	Fingolimod	40	64.73 ± 15.01	64	39.8–94.0	
Role Limitations due to Emotional Problems	Fingolimod	40	19.25 ± 7.41	24	0–24	
Emotional Well-being	Fingolimod	40	18.66 ± 4.45	17	11.6–29.0	
Health Distress (CMH)	Fingolimod	40	7.10 ± 2.05	6.6	2.2–11	
Cognitive Function	Fingolimod	40	6.88 ± 3.45	6	2.2–13.5	
Overall Quality of Life	Fingolimod	40	12.80 ± 3.36	13.2	5.1–18.0	

* *p* < 0.05 was considered statistically significant.

## Data Availability

All details about the study can be obtained from the corresponding author.

## Abbreviations

(CNS)	Central nervous system
(MS)	Multiple sclerosis
(QoL)	Quality of life
(DMTs)	Disease-modifying therapies
(RRMS)	Relapsing-remitting MS
(HRQoL)	Health-related QoL
(EDSS)	Expanded Disability Status Scale
(HDRS)	Hamilton Depression Rating Scale
(HARS)	Hamilton Anxiety Rating Scale
(MSQOL-54)	Multiple Sclerosis QoL-54
(CMH)	Composite Mental Health
(PROs)	Patient-reported outcomes
(CPH)	Composite Physical Health
